# Diagnosis, Severity, and Prognosis from Potential Biomarkers of COVID-19 in Urine: A Review of Clinical and Omics Results

**DOI:** 10.3390/metabo14120724

**Published:** 2024-12-22

**Authors:** Jennifer Narro-Serrano, Frutos Carlos Marhuenda-Egea

**Affiliations:** 1 Department of Physical Chemistry, University of Alicante, 03690 Alicante, Spain; jennifer.narro@ua.es; 2 Department of Biochemistry and Molecular Biology and Soil Science and Agricultural Chemistry, University of Alicante, 03690 Alicante, Spain

**Keywords:** COVID-19, urine, biomarkers, diagnosis, severity, prognosis, clinical, omics

## Abstract

The COVID-19 pandemic, caused by the SARS-CoV-2 virus, has spurred an extraordinary scientific effort to better understand the disease’s pathophysiology and develop diagnostic and prognostic tools to guide more precise and effective clinical management. Among the biological samples analyzed for biomarker identification, urine stands out due to its low risk of infection, non-invasive collection, and suitability for frequent, large-volume sampling. Integrating data from omics studies with standard biochemical analyses offers a deeper and more comprehensive understanding of COVID-19. This review aims to provide a detailed summary of studies published to date that have applied omics and clinical analyses on urine samples to identify potential biomarkers for COVID-19. In July 2024, an advanced search was conducted in Web of Science using the query: “covid* (Topic) AND urine (Topic) AND metabol* (Topic)”. The search included results published up to 14 October 2024. The studies retrieved from this digital search were evaluated through a two-step screening process: first by reviewing titles and abstracts for eligibility, and then by retrieving and assessing the full texts of articles that met the specific criteria. The initial search retrieved 913 studies, of which 45 articles were ultimately included in this review. The most robust biomarkers identified include kynurenine, neopterin, total proteins, red blood cells, ACE2, citric acid, ketone bodies, hypoxanthine, amino acids, and glucose. The biological causes underlying these alterations reflect the multisystemic impact of COVID-19, highlighting key processes such as systemic inflammation, renal dysfunction, critical hypoxia, and metabolic stress.

## 1. Introduction

The COVID-19 pandemic, caused by the SARS-CoV-2 virus, has driven a remarkable scientific effort to better understand the pathophysiology of the disease and to develop diagnostic and prognostic tools that can guide more precise and effective clinical management [1,2]. Biomarker identification, in particular, has emerged as a key area of focus, offering critical insights into the presence, progression, and severity of the disease [3]. Given the heterogeneity of COVID-19, which impacts multiple physiological systems and presents variable clinical responses [4,5], biomarkers provide an invaluable means for early detection, continuous monitoring, and the differentiation of disease severity, as well as for anticipating potential complications. Furthermore, biomarkers provide a window into the molecular mechanisms underlying the SARS-CoV-2 infection and the host immune response, which is key to the development of targeted therapies and more personalized treatment approaches [6].

Currently, the reference method for diagnosing COVID-19 remains RT-PCR, which detects viral RNA in respiratory tract samples with a high sensitivity and specificity [7]. This technique is complemented by rapid antigen tests, which, although less sensitive, offer faster results and the convenience of self-administration [7]. Serological tests, detecting antibodies such as IgM and IgG, are useful for identifying past or recent infections [8], while radiological imaging techniques, including computed tomography and chest X-rays, are critical for assessing pulmonary damage caused by the disease [9]. These diagnostic tools, when combined, form the foundation for the early identification and clinical management of COVID-19 cases.

The clinical severity of COVID-19 ranges widely, from asymptomatic or mild cases to severe and critical conditions [10]. Clinical evaluation, including symptoms such as dyspnea, tachypnea, and reduced oxygen saturation levels, remains central to determining disease severity and guiding appropriate interventions [2]. Laboratory findings, such as lymphopenia and elevated blood biomarkers like D-dimer, C-reactive protein, and ferritin, provide additional insights into disease progression and inflammatory activity [11]. Factors such as an advanced age, underlying comorbidities, and an exaggerated inflammatory response are consistently associated with worse outcomes [12]. In intensive care settings, clinical scoring systems, such as the Sequential Organ Failure Assessment (SOFA) index, are routinely employed to predict mortality and guide treatment priorities [13].

The management of COVID-19 is tailored according to disease severity [2]. Mild cases are managed conservatively on an outpatient basis, focusing on rest, hydration, and symptomatic control. Moderate cases often require oxygen therapy, with antivirals such as remdesivir administered in appropriate clinical settings to reduce viral replication during the early phases of infection [2]. For severe and critical cases, the treatment strategies include supplemental oxygen, mechanical ventilation, corticosteroids like dexamethasone to mitigate systemic inflammation, and anticoagulant therapy to prevent thromboembolic complications [2]. Immunomodulatory therapies, such as IL-6 inhibitors, are utilized to manage severe hyperinflammatory responses [2]. Following recovery from the acute phase, pulmonary rehabilitation, physical therapy, and psychological support are essential components of care to address the long-term sequelae observed in many patients [2].

While progress has been made in COVID-19 diagnostics and prognostics, challenges persist in improving their precision and accessibility. The heterogeneity of the disease complicates efforts to establish universally reliable biomarkers for early detection, risk stratification, and long-term monitoring. Ongoing research is, therefore, increasingly focused on the integration of innovative approaches to overcome these limitations. In this regard, urine has emerged as a particularly promising biological sample for biomarker discovery, offering several practical advantages over other biofluids. Its non-invasive and low-risk collection method enables frequent and large-volume sampling, making it ideal for longitudinal studies and routine monitoring [14]. These characteristics position urine as a valuable alternative for identifying biomarkers that could play a critical role in COVID-19 diagnosis and prognosis. However, urine composition is inherently variable, influenced by factors such as hydration status, dietary habits, physical activity, medication use, and individual health conditions. These variables underscore the importance of implementing standardized protocols and rigorous analytical methods to ensure the reliability and reproducibility of urine-based diagnostics [15]. Despite these challenges, urine remains a readily accessible and underutilized resource with considerable potential to advance biomarker research and facilitate the development of non-invasive diagnostic and monitoring tools for both acute and long COVID.

The integration of omics data with standard biochemical analyses offers a deeper and more holistic understanding of the disease [16]. Omics technologies provide a comprehensive overview of molecular alterations associated with infection and disease progression, while standard biochemical analyses contribute established, quantifiable clinical parameters that reflect the patient’s physiological state. The combination of these approaches enables the correlation of molecular changes with clinical outcomes, facilitating the identification of robust and relevant biomarkers for diagnosis and prognosis. This synergy is particularly valuable in the context of COVID-19, where the disease’s multisystem impact generates complex biological responses reflected across diverse molecular and biochemical profiles [17].

This review aims to provide a comprehensive overview of studies that have applied omics and clinical analyses to urine samples for the identification of potential COVID-19 biomarkers. By examining key findings from both clinical and omics studies, we emphasize methodological advancements, highlight potential biomarkers capable of assessing disease severity, aiding diagnosis, or offering prognostic insights, and address current limitations requiring further investigation. This review serves as a comprehensive resource for researchers and clinicians interested in exploring non-invasive monitoring tools for COVID-19 and deepening their understanding of the mechanisms underlying the variability in responses to the SARS-CoV-2 infection.

## 2. Methods

The PRISMA statement guidelines were followed during the reporting of this study [18].

### 2.1. Eligibility Criteria

We included all experimental studies comparing findings from metabolomics, lipidomics, proteomics, transcriptomics, or clinical analyses performed on human urine samples. The analyses categorized under the term “clinical analyses” include the evaluation of chemical parameters—such as the detection of glucose, proteins, ketones, and blood, using reagent strips or automated methods—and microscopic parameters, involving the examination of urinary sediment to identify cells, crystals, or casts, all conducted under standardized conditions. Additionally, specialized tests focused on the measurement of specific compounds, including metabolites, electrolytes, metals, or proteins, were also considered. The only criterion for inclusion was that these analyses were performed within a hospital or clinical setting. Furthermore, only parameters that showed statistically significant differences between the compared groups were included in this review.

Only studies involving human subjects or datasets were included, ensuring that the analysis was based on real-world human data rather than experimental models. Although some of the articles included in the review contained information derived from surveys or analyses of other biological matrices, only data obtained from urine sample analyses were considered. Reviews, editorials, conference summaries, and communications articles were excluded.

### 2.2. Search Strategy

In July 2024, an advanced search was conducted in Web of Science (Clarivate Analytics, Philadelphia, PA, USA) with the query: “covid* (Topic) AND urine (Topic) AND metabol* (Topic)”. The results that appeared until 14 October 2024 were included. 

The retrieved studies from the digital search were evaluated in a two-step screening process. First, titles and abstracts were reviewed to identify eligible articles. Next, the full texts of these articles were retrieved and assessed based on specific inclusion criteria. Endnote (Clarivate Analytics, Philadelphia, PA, USA) was used to remove the duplicate articles. 

### 2.3. Data Extraction

Data were collected using a pre-designed extraction sheet. The following information was gathered: the study title, abstract, article type, publication date, methodological approach and analytical techniques used, characteristics of the study population, and potential biomarkers identified for diagnostic, severity, or prognostic purposes. 

## 3. Results

### 3.1. Search Results

The initial search retrieved a total of 913 studies. Six duplicate articles, which were preprints of already published articles, were removed using EndNote, leaving 907 articles eligible for title and abstract screening. Following this first screening phase, 840 articles were excluded, as they did not meet the inclusion criteria (i.e., experimental studies in metabolomics, lipidomics, proteomics, transcriptomics, or clinical analysis; analyzing urine samples; and involving human-derived samples). Consequently, the full texts of 67 articles were retrieved for the second screening phase. 

**Figure 1:**
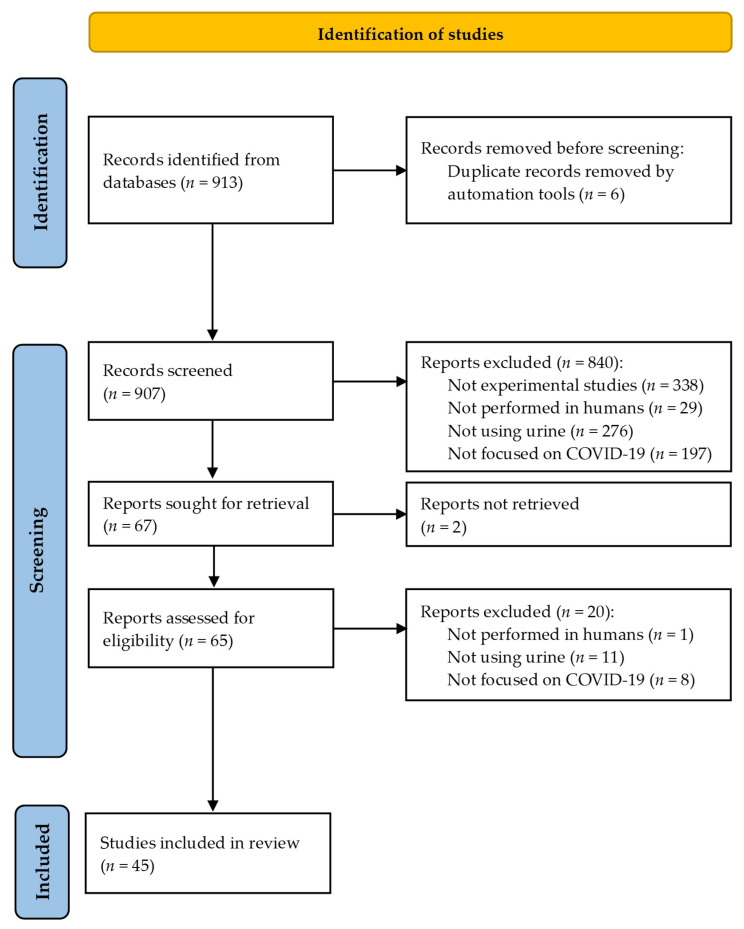
PRISMA Flow chart.

During this phase, two articles were eliminated due to the inability to access their full texts. After thoroughly reviewing the remaining articles, 20 were excluded for not meeting the inclusion criteria. Ultimately, 45 articles were included in this study. Figure 1 provides a detailed flow diagram of the article selection process.

### 3.2. Characteristics of the Included Studies

Forty-five studies were included in our review. Detailed characteristics of the included studies are outlined in Table 1.

## 4. Discussion

### 4.1. General Review of the Results

Out of the initial 913 articles retrieved, a two-step screening process was employed to select those meeting the inclusion criteria. First, the titles and abstracts were assessed to identify potentially eligible studies, and then, full texts were reviewed in detail. Following this filtering, only 45 specifically focused on the identification of urinary biomarkers in COVID-19 patients. Most excluded studies concentrated on other biofluids, particularly blood serum. Interestingly, many articles retrieved during the initial search—especially those published between 2020 and 2021—were unrelated to COVID-19, despite being tagged with the keyword in their metadata. This likely reflects an effort by some authors to increase the visibility of their work in search engines by including a highly trending keyword at the time, even if their research had no substantive connection to the disease.

**Figure 2:**
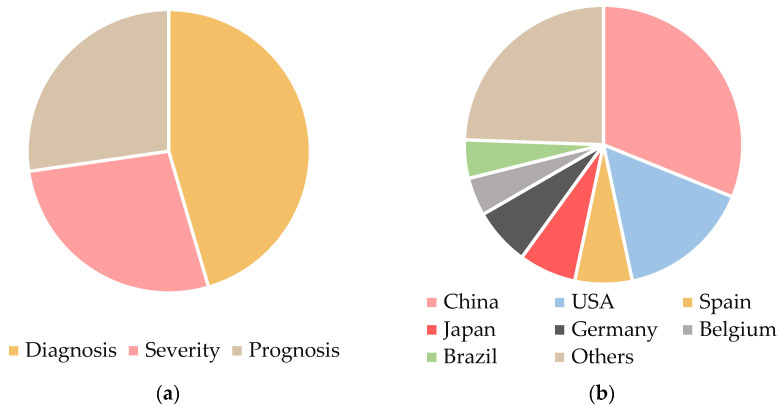
Pie charts depicting (**a**) the distribution of publications identifying biomarkers for COVID-19, categorized by their intended purpose: diagnosis (30), severity (18), and prognosis (18); and (**b**) the geographic distribution of COVID-19 biomarker studies, with contributions from China (14), the USA (7), Spain (3), Japan (3), Germany (3), Belgium (2), Brazil (2), and a combined category labeled “Others,” which includes countries with a single publication each: Austria, Bangladesh, Canada, Czech Republic, Denmark, Iran, Portugal, Saudi Arabia, Slovakia, Sweden, and Turkey.

The findings from the studies included in this review are summarized in Table 1. Figure 2a illustrates the distribution of articles based on the purpose of the identified biomarkers. The majority of studies focus on diagnostic biomarkers, with 30 out of 45 articles (67%) addressing this objective. Studies targeting severity and prognosis are equally represented, with each accounting for 18 articles (40%).

Figure 2b presents the geographic distribution of COVID-19 biomarker studies. Asia leads with 20 publications (45%), predominantly from China, reflecting a significant contribution from regions where the pandemic first emerged. However, this also raises concerns about the potential overrepresentation of Asian populations in biomarker research. Such an overrepresentation could result in findings that are more reflective of population-specific characteristics—such as genetic, environmental, or healthcare-related factors—potentially limiting their applicability to other regions.

Europe follows with 15 articles (33%), while the Americas contribute 10 articles (22%). Of these, the majority are from North America. The relatively low representation of studies from other regions, such as Africa and Oceania, underscores the need for future research to include more diverse cohorts. Expanding geographic and demographic diversity in biomarker studies would enhance the generalizability of findings across different populations, ensuring that identified biomarkers are robust and effective in a global clinical context.

There is considerable heterogeneity among the potential biomarkers identified, both between and within analytical techniques. Even studies using the same approach show variations, reflecting the complex systemic response to the SARS-CoV-2 infection. This response involves processes such as inflammation, oxidative stress, metabolic disruptions, and multi-organ dysfunction. Additionally, each analytical approach relies on distinct detection principles, leading to the identification of different biomarkers and providing unique insights into the disease’s pathophysiology. 

These methodological variations are further compounded by discrepancies in pre-analytical phases, sample preparation, and validation strategies. For instance, several studies [5,23] used urine samples collected under clinical conditions without stringent standardization, while others [37] followed rigorous protocols, including freezing samples at −80 °C immediately after collection to ensure stability. Such differences significantly affect the integrity of sensitive biomarkers. Additionally, the preparation methods ranged from basic centrifugation [38] to advanced techniques like ultrafiltration and derivatization [37,45], which enhance the detection of low-abundance analytes.

According to Table 1, metabolomics based on liquid chromatography is the most commonly used technique. Specifically, metabolomics was used in 20 studies, clinical analyses in 16, and proteomics in 8. Other techniques, such as lipidomics and transcriptomics, were utilized in two studies each. The reliance on metabolomics reflects its ability to detect subtle biochemical changes, as highlighted in studies like Baiges-Gaya et al. [49] and Marhuenda-Egea et al. [43]. Clinical analyses, on the other hand, were more prevalent in the early months of the pandemic, likely due to their speed and cost-effectiveness. Studies such as Temiz et al. [36] relied on standard parameters like creatinine and albumin levels, which, while reproducible, do not uncover novel biomarkers.

In contrast, proteomics, as demonstrated by Bi et al. [29] and Chen et al. [35], allow for the identification of new proteins and small molecules involved in inflammatory and immune responses. However, the results from these approaches are highly dependent on the analytical platforms used (e.g., LC-MS/MS, ^1^H NMR, and GC-MS) and the experimental conditions. For instance, studies using lipidomics, such as Kurano et al. [45], offer valuable insights into sphingolipids and glycerophospholipids’ role in COVID-19-associated kidney injuries, and yet, the variability in sample preparation limits the cross-study comparability. Similarly, Robertson et al. [39] innovated with Raman spectroscopy, which, although promising, remains underexplored due to its limited application in urinary biomarkers.

The methodological differences extend to the statistical approaches and validation strategies employed. Advanced multivariate analyses, such as PCA and regression models, were used in studies like Li et al. [26], Marhuenda-Egea et al. [43], and Baiges-Gaya et al. [49] to identify patterns and correlate biomarkers with disease progression. However, simpler methods, such as ANOVA and t-tests, were favored in studies like Zeng et al. [24] and Werion et al. [23], which focus on established clinical parameters rather than exploratory biomarkers. The validation of findings was another area of disparity; while studies such as Marhuenda-Egea et al. [43] validated their results in independent cohorts, others, like Li et al. [26], relied primarily on technical replicates, limiting their generalizability.

Transcriptomics, as seen in studies like Soltane et al. [56], provide in-depth insights into gene regulation and inflammatory processes. However, they remain less reproducible due to the variability in sample handling and the limited number of studies employing this technique for urinary biomarkers in COVID-19. This variability highlights the need for standardization across laboratories, not only in pre-analytical and analytical methods but also in data processing and interpretation.

### 4.2. Diagnosis

The selected potential diagnostic biomarkers are presented in Table 2. Data from studies comparing COVID-19 patients against three distinct groups have been considered: healthy controls, patients with non-COVID-19 pathologies, and asymptomatic COVID-19 patients. Biomarkers were selected if they were identified in three or more studies showing consistent behavior (either increased or decreased concentration in COVID-19 patients compared to the control group).

The potential diagnostic biomarkers identified (Table 2) encompass both metabolites—such as citric acid, amino acids, ketone bodies, and kynurenine—and proteins, including total protein levels and ACE2.

The changes summarized in Table 2 reflect a profound disruption of energy metabolism, a severe catabolic state, and multisystem damage, particularly affecting the kidneys (proteinuria and aminoaciduria). Alterations in the Krebs cycle (low citric acid) and lipid metabolism (elevated ketone bodies) are compounded by markers of inflammation and immunosuppression (elevated kynurenine).

Citric acid is a key intermediate in the Krebs cycle, essential for ATP production in mitochondria. In COVID-19 patients, severe inflammation and mitochondrial hypoxia impair the Krebs cycle’s efficiency, leading to reduced citric acid levels in the urine [43]. This reduction may also result from renal dysfunction affecting the filtration and reabsorption of citric acid. Low urinary citric acid, therefore, reflects both mitochondrial dysfunction and potential renal impairment. Proteinuria indicates glomerular damage, likely mediated by the virus’s interaction with ACE2 receptors in renal cells [36]. Systemic inflammation and endothelial damage further compromise the glomerular barrier, allowing proteins to pass into the urine. Aminoaciduria reflects proximal tubular damage, which impairs amino acid reabsorption [23]. The loss of essential amino acids affects protein synthesis and reduces antioxidant capacity, such as the production of glutathione, exacerbating oxidative stress.

The angiotensin-converting enzyme 2 (ACE2) receptor is the primary entry point for SARS-CoV-2 into human cells [63]. Its widespread expression in key tissues, including the lungs, kidneys, intestine, and heart, makes it a central player in COVID-19 pathophysiology. Increased ACE2 presence in urine may result from direct viral action targeting ACE2 in proximal tubular epithelial cells, systemic inflammation triggered by the viral infection causing widespread tissue damage, or compensatory regulation. As SARS-CoV-2 reduces ACE2 expression in infected tissues, the body may compensate by increasing soluble ACE2 synthesis in unaffected tissues [63].

The elevated production and excretion of ketone bodies in COVID-19 patients are linked to tissue hypoxia; COVID-19-induced hypoxia reduces oxygen availability for oxidative phosphorylation, redirecting energy metabolism toward ketogenesis [64]. Inflammatory and metabolic stress associated with COVID-19 causes insulin resistance and decreases glucose availability as an energy source. This is exacerbated in patients with diabetes or stress-induced hyperglycemia. Additionally, impaired tubular function may reduce the reabsorption of ketone bodies, increasing their urinary excretion.

These metabolic and renal biomarkers—low citric acid, proteinuria, aminoaciduria, elevated ACE2, and ketone bodies—highlight the complex interplay of mitochondrial dysfunction, inflammation, and renal impairment in COVID-19. Each provides unique insights into the disease’s pathophysiology: low citric acid reflects mitochondrial hypoxia and impaired energy production, proteinuria and aminoaciduria signal glomerular and tubular damage, urinary ACE2 levels underscore the virus’s direct impact on target organs, and ketone bodies reveal metabolic stress and adaptive shifts in energy utilization. The integration of these potential biomarkers into diagnostic workflows could improve the early detection and monitoring of COVID-19, particularly in understanding its systemic and renal impacts.

### 4.3. Severity

In the severity analysis, comparisons were made among mild, moderate, and critical cases. The classification of cases was based on the WHO severity scale [10]: critical (stages 6–8), moderate (stages 4–5), mild (stages 1–3), and asymptomatic (COVID-19 patients without any symptoms). Due to the limited number of studies investigating severity, biomarkers were selected if they were identified in two or more studies showing consistent behavior. Potential predictive biomarkers of severity are detailed in Table 3.

The potential biomarkers for disease severity (Table 3) include metabolites, such as glucose and ketone bodies, total protein levels, and cellular components, specifically red blood cells.

The biomarkers listed in Table 3 reflect a more advanced systemic damage associated with multi-organ dysfunction and progression to critical states. Glucosuria in COVID-19 patients results from worsening glucose metabolism [65], as already indicated by diagnostic biomarkers, driven by inflammation, hypoxia, insulin resistance, and tubular dysfunction. Proteinuria and hematuria reflect severe glomerular and tubular damage caused by inflammation, hypoxia, and coagulopathies [66]. Persistent elevations of ketone bodies indicate an extreme metabolic adaptation to hypoxia and energy stress.

### 4.4. Prognosis

Table 4 presents the potential predictive biomarkers of prognosis, which aid in identifying patients at elevated risk of adverse outcomes, including mortality and specific complications such as acute kidney injury (AKI) or long-COVID-19 syndrome. Given the limited number of studies focused on prognosis, biomarkers were included only if they were identified in two or more studies with consistent findings.

The potential prognostic biomarkers (Table 4) are exclusively metabolites, including ketone bodies, kynurenine, the kynurenine-to-tryptophan ratio (KTR), neopterin, and hypoxanthine.

Elevated levels of neopterin, kynurenine, and the kynurenine/tryptophan ratio (KTR) are interconnected and reflect sustained immune activation and chronic inflammation. These biomarkers are driven by macrophage activation and the induction of interferon-gamma (IFN-γ), a key cytokine in the exacerbated immune response. The kynurenine pathway is activated by the enzyme indoleamine 2,3-dioxygenase (IDO1), whose activity increases in response to proinflammatory cytokines, particularly IFN-γ [67]. This activation has multiple physiological consequences. First, the accelerated conversion of tryptophan into kynurenine reduces the availability of tryptophan for other essential processes, such as serotonin synthesis, affecting both neuro-metabolic balance and immune function. Additionally, kynurenine and its metabolites exhibit significant immunomodulatory effects, including T-cell inhibition and the promotion of an immunosuppressive state, contributing to a dysregulated immune response that increases susceptibility to secondary infections and severe complications. Furthermore, these metabolites generate oxidative stress, exacerbating tissue damage in organs such as the kidneys and nervous system. This process worsens tubular and glomerular damage, increasing the risk of AKI during the acute phase and persisting throughout recovery, thereby contributing to prolonged symptoms associated with long-COVID syndrome [67].

Hypoxanthine is a key metabolite in purine catabolism, and its urinary levels reflect the balance between its production during cellular stress and its subsequent metabolism [50]. In the context of COVID-19, urinary hypoxanthine levels are influenced by various metabolic and physiological factors that vary according to the stage of the disease, particularly in patients with worse outcomes. During the initial weeks of infection, patients with poor prognoses often exhibit low urinary hypoxanthine levels [50]. Hypoxanthine, accumulated intracellularly due to oxygen deprivation, is diverted toward the purine salvage pathway or converted to urate via xanthine oxidase. In early hypoxic states, hypoxanthine metabolism prioritizes urate production, potentially reducing its excretion in urine. This also reflects metabolic overload in tissues, where hypoxanthine is rapidly metabolized in an attempt to maintain energy homeostasis.

However, in later stages of the disease, patients with worse outcomes tend to show higher urinary levels of hypoxanthine [50]. This increase can be attributed to the progressive collapse of adaptive mechanisms against hypoxia. In critical states, massive hypoxanthine release occurs from damaged tissues due to cell death and systemic energy depletion. Additionally, xanthine oxidase activity may be insufficient or altered in these patients, leading to hypoxanthine accumulation and increased urinary excretion. This transition from low to high levels reflects the progression of the disease from an initial compensatory phase to a state of generalized metabolic dysfunction and advanced tissue damage.

On the other hand, increasingly elevated levels of ketone bodies indicate an adaptive metabolic shift toward ketogenesis, triggered by hypoxia, insulin resistance, and glucose depletion as the primary energy source [64]. This elevation reflects a state of extreme metabolic stress and the body’s attempt to maintain energy homeostasis under critical conditions. Both urinary biomarkers—hypoxanthine and ketone bodies—are indicative of severe metabolic imbalance and the body’s failed effort to adapt to the conditions imposed by the infection, making them valuable tools for assessing disease severity and the risk of adverse outcomes.

### 4.5. Limitations

The main limitation of this review is the significant heterogeneity in the findings. Studies vary widely in their methodologies, employing diverse techniques, analytical platforms, and study populations, which makes direct comparison of results challenging. Furthermore, there is no consensus on reference values or clinical thresholds for many of the proposed biomarkers, limiting their translation into clinical practice.

Another key issue is that most studies are conducted on small sample sizes, often focusing on specific cohorts such as hospitalized or critically ill patients. This leaves out important subpopulations, including outpatients, pediatric cases, or asymptomatic individuals, raising concerns about the generalizability of the identified biomarkers to the broader population affected by COVID-19. Moreover, many of the included populations have multiple comorbidities, which may further confound the interpretation of biomarker specificity for COVID-19.

A further potential confounding factor in comparing results across studies is the lack of information in most articles regarding the specific SARS-CoV-2 strain infecting the COVID-19 patients. Given that different viral strains have been shown to produce distinct symptomatology [68], it is plausible that they also result in differing alterations detectable in urine, complicating the interpretation and harmonization of biomarker findings.

To address these limitations, future research must prioritize the standardization of protocols to enhance reproducibility and comparability across studies. Developing international guidelines for sample collection, preparation, and analysis, as well as establishing reference values and clinical thresholds for each biomarker, will be critical for their clinical implementation. Additionally, larger, more representative studies that include diverse populations—such as outpatients, pediatric groups, and vulnerable individuals—are essential to ensure the biomarkers are broadly applicable. It will also be important to include populations with other inflammatory, viral, or metabolic diseases to identify biomarkers specific to COVID-19 and rule out potential confounding effects from comorbidities. Furthermore, validating promising biomarkers through prospective clinical studies across various contexts, such as hospital and community settings, will solidify their relevance. Finally, exploring the utility of multi-biomarker panels that integrate pathways related to immunity, metabolism, and tissue damage may provide a more robust diagnostic and prognostic tool, offering an improved sensitivity and specificity for COVID-19.

## 5. Conclusions

The biological mechanisms underlying the selected biomarkers reflect the multisystemic impact of COVID-19. The identified potential diagnostic biomarkers, including reduced citric acid levels and elevated levels of proteins, amino acids, ACE2, and ketone bodies, highlight early metabolic and renal alterations associated with mitochondrial dysfunction, inflammation, and energy stress. As the disease progresses, findings such as glucosuria, proteinuria, hematuria, and elevated ketone bodies indicate the onset of severe complications. Potential prognostic biomarkers such as kynurenine, KTR, neopterin, and hypoxanthine reflect a combination of persistent immune activation, chronic inflammation, and oxidative stress that may shape long-term outcomes.

Urinary biomarkers therefore represent a promising non-invasive tool for diagnosing COVID-19, evaluating disease severity, and informing prognosis. Future studies should focus on standardizing protocols, expanding the range of clinical settings and populations assessed, and validating these findings in larger cohorts. Integrating these potential biomarkers into multidimensional panels could enhance both the sensitivity and specificity of COVID-19 diagnosis and management.

## Figures and Tables

**Table 1:** Characteristics of the included studies. Population data nomenclature has been standardized to facilitate data analysis; refer to the Appendix A for further details. (↑): statistically significant increased compounds, with *p*-value < 0.05. (↓): statistically significant decreased compounds, with *p*-value < 0.05. (*): specific states and differentiators of the population, such as certain physiological conditions or specific diseases.

Population	Approach and Analytical Platform	Results and Potential Biomarkers	Reference
HC (*n* = 141) COVID-19 (*n* = 141)	Metabolomics (DSI-MS)	**Diagnosis (COVID-19 vs. HC)** ↑: styrene, diethanolamine, butanone, norcotinine, 3-tert-butyl-4-hydroxyanisole, caproic acid, homocysteine, 2-aminobenzoic acid, 6-hydroxynicotinic acid, desaminotyrosine, glycylproline, citrulline, 4-methoxybenzenepropanoic acid, 3-methoxybenzenepropanoic acid, (3-hydroxyphenyl)hydracrylic acid, 4-pyridoxic acid, and l-cysteine	[19] 28 February 2020
COVID-19 (*n* = 93) - moderate (*n* = 22) - critical (*n* = 71)	Clinical analysis	**Severity (Critical vs. Moderate)** ↑: proteinuria, hematuria, and glucosuria.	[20] 7 March 2020
COVID-19 (*n* = 71) - moderate (*n* = 32) - critical (*n* = 21)	Clinical analysis	**Severity (Critical vs. Moderate)** ↑: Glucose, ketone bodies, red blood cells, and white blood cells	[21] 7 April 2020
COVID-19 * (*n* = 16) * pregnant women	Clinical analysis	**Diagnosis (COVID-19 vs. Reference range)** ↑: ketone bodies	[22] 22 April 2020
COVID-19 (*n* = 49)	Clinical analysis	**Diagnosis (COVID-19 vs. Reference range)** ↑: low-molecular-weight proteinuria, aminoaciduria, phosphaturia, and uricosuria	[23] 8 May 2020
COVID-19 (*n* = 138) - mild (*n* = 70) - moderate (*n* = 68)	Clinical analysis	**Severity (Moderate vs. Mild)** ↑: Proteinuria, chromium, manganese, copper, cadmium, and selenium ↓: Creatinine, arsenic, and thallium **Prognosis (Non-survivor vs. Survivors)** ↑: Chromium, manganese, copper, cadmium, mercury, and lead	[24] 13 June 2020
COVID-19 (*n* = 48) - critical (*n* = 48)	Clinical analysis	**Diagnosis (COVID-19 vs. Reference range)** ↑: Microalbuminuria **Prognosis (Critical vs. Reference range)** ↑: Develop acute kidney injury (AKI)	[5] 18 June 2020
HC (*n* = 4) COVID-19 (*n* = 4)	Proteomics (Luminex)	**Diagnosis (COVID-19 vs. HC)** ↑: Inflammatory cytokines (IL-6, IL-8)	[25] 6 August 2020
COVID-19 (*n* = 6) - mild (*n* = 3) - moderate (*n* = 3)	Proteomics (LC-MS/MS)	**Severity (Moderate vs. Mild)** ↑: complement activation (IGLV1–40, IGLV1–44, C3, and C4B), immune response (IGLV1–40, IGLV1–44, C3, and FKBP1A), oxidant detoxification (TXNDC17, FABP1, and PARK7), response to hypoxia (LMNA, HYOU1, and FABP1), and oxidative stress-induced apoptosis (SOD1; PARK7). ↓: platelet degranulation (PSAP, EGF, FGA, CD9, CLEC3B, and LGALS3BP), glucose metabolism (GNS, NAGLU, GLB1, HSPG2, YWHAZ, ALDOB, TPI1, RBP4, and PGK1), protein metabolism (MME, CPM, HSPG2, and NAPSA), and lipid metabolic and transport (LRP2, RBP4, HSPG2, APP, CUBN, APOA1, and NPC2)	[26] 23 December 2020
non-COVID-19 * (*n* = 6) COVID-19 (*n* = 9) * flu-like symptoms	Metabolomics (UPLC-MS/MS)	**Diagnosis (COVID-19 vs. non-COVID-19)** ↑: ornithine, 8-propyloxycaffeine, and taurine	[27] 14 February 2021
HC (*n* = 5086) COVID-19 (*n* = 557)	Clinical analysis	**Diagnosis (COVID-19 vs. HC)** ↑: International normalized ratio (INR) ↓: pH	[28] 9 March 2021
HC (*n* = 27) COVID-19 (*n* = 71) - moderate (*n* = 48) - critical (*n* = 23)	Metabolomics and Proteomics (UPLC-MS/MS)	**Diagnosis (COVID-19 vs. HC)** ↑: Proteinuria, ROS metabolites (quinolinate), 3-hydroxyanthranilate, fucose, urate and kynurenine ↓: ROS metabolites (*N*-acetylcysteine), cytokines (CXCL14, IL-34, CCL14), tryptamine, serotonin, citrate, and ESCRT **Severity (Critical vs. Moderate)** ↓: CUBN, RHOA, RAC1, cytokines (CXCL14, IL-34, and CCL14), adenosine, tryptamine, serotonin, and antioxidant enzymes (SOD3, GPX4). Also, EphB receptors (EPHB2, EPHB3, EPHB4, and EPHB6), SIPR3, RAMP3, and EGF	[29] 14 April 2021
HC (*n* = 11) COVID-19 (*n* = 25) - mild (*n* = 15) - critical (*n* = 10)	Transcriptomics (ddPCR)	**Diagnosis (COVID-19 vs. HC)** ↑: miR-2392 (related to inflammation and glycolysis) **Severity (Critical vs. Mild)** ↑: miR-2392 (related to inflammation and glycolysis)	[30] 23 April 2021
non-COVID-19 * (*n* = 1438) COVID-19 (*n* = 119) - mild (*n* = 66) - critical (*n* = 53) * Patients dataset of diabetic kidney disease, AKI, and heart failure	Proteomics (CE-MS)	**Diagnosis (COVID-19 vs. non-COVID-19)** ↓: fragments of CD99 and PIGR **Severity (Critical vs. Mild)** ↓: fragments of CD99, PIGR, different types of collagens, α-1-antitrypsin, β-2-microglobulin, fibrinogen, and protein S100-A9	[31] 23 May 2021
HC (*n* = 336) COVID-19 (*n* = 113)	Clinical analysis	**Diagnosis (COVID-19 vs. HC)** ↑: magnesium and oxalate ↓: urea, sodium, potassium, uric acid, and citrate	[32] 8 June 2021
COVID-19 (*n* = 68) - moderate (*n* = 27) - critical (*n* = 41) Repeated at days: 1–2, 3–4, 5–7, and up to 25	Proteomics (ELISA)	**Severity (Dead vs. Survivors)** ↑: LAG-3 and IL-2 **Severity (Critical vs. Moderate)** ↑: IL-12 ↓: CAIX, CCL23, Gal-9, HGF, HO-1, IL-15, IL-18, MCP-1, MCP-3, MUC-16, PD-L1, TNFRS12A, and TNFRS21	[33] 8 July 2021
COVID-19 (*n* = 24) COVID-19 * (*n* = 22) * AKI	Metabolomics (LC-MS/MS)	**Prognosis (COVID-19 AKI vs. COVID-19)** ↑: quinolinate/tryptophan ratio, kynurenic acid, and glucuronic acid ↓: metabolites related to purines (xanthine, hypoxanthine, xanthosine, adenosine, cyclic AMP, urate, *N*-acetylaspartate, *N*,*N*-dimethylarginine, and histidine), TCA cycle (pantothenate, trans-aconitate, methylglutarate, and tyrosine), and NAD^+^ metabolism (nicotinamide, tryptophan, *N*-α-acetyl-lysine, and 3-hydroxyanthranilic acid)	[34] 19 August 2021
Cohort 1: HC (*n* = 36) COVID-19 (*n* = 70) - asymptomatic (*n* = 70) Cohort 3: HC (*n* = 10) COVID-19 (*n* = 14)	Proteomics (LC-MS)	**Diagnosis (Asymptomatic vs. HC)** ↑: proteins involved in neutrophil- and leukocyte-mediated immunity (STAT3, CTNNB1, TGFB1, COPB1, PSMD12, MYD88, NCK1, XRCC5, and DDOST) ↓: proteins involved in reverse cholesterol transport (VEGFA and APOA5) **Diagnosis (COVID-19 vs. HC)** ↓: proteins involved in reverse cholesterol transport (LIPG and EHHADH)	[35] 10 September 2021
HC (*n* = 11) COVID-19 (*n* = 36) - mild/moderate (*n* = 24) - critical (*n* = 12)	Clinical analysis	**Diagnosis (COVID-19 vs. HC)** ↑: micro-hematuria and proteinuria **Severity (Critical vs. Moderate)** ↑: proteinuria and KIM-1/creatinine ratio **Prognosis (Non-survivor vs. Survivors)** ↑: proteinuria and KIM-1/creatinine ratio	[36] 13 September 2021
HC (*n* = 176) non-COVID-19 * (*n* = 55) COVID-19 (*n* = 86) - mild (*n* = 1) - moderate (*n* = 74) - critical (*n* = 11) * Pneumonia	Proteomics (HPLC MS/MS)	**Diagnosis (COVID-19 vs. HC)** ↑: proteins involved in the SARS-CoV2 infection (ACE2 DDX58/RIG-I), and interferon signaling (STAT1) **Diagnosis (COVID-19 vs. non-COVID-19)** ↑: proteins involved in the SARS-CoV2 infection (ACE2) **Severity (Critical vs. Moderate)** ↑: CD14, RBP4, SPON2, GMFG, SERPINA1, SERPINB6, SERPINC1, TTR, RAB3A, and SAA2	[37] 15 September 2021
non-COVID-19 * (*n* = 78) COVID-19 * (*n* = 88) - mild (*n* = 23) - moderate (*n* = 45) - critical (*n* = 20) * Acute renal injuries	Clinical analysis	**Diagnosis (COVID-19 vs. non-COVID-19)** ↓: granular casts, epithelial casts, waxy casts, and urinary levels of total protein, microalbumin, *N*-acetyl-β-D-glucosaminidase, α1-microglobulin, liver-type fatty acid-binding protein, and neutrophil gelatinase-associated lipocalin **Severity (Moderate vs. Mild)** ↑: Red blood cell particles, *N*-acetyl-β-D-glucosaminidase, α1-microglobulin, liver-type fatty acid-binding protein, and neutrophil gelatinase-associated lipocalin **Severity (Critical vs. Moderate)** ↑: hyaline casts, granular casts, epithelial casts, and urinary levels of *N*-acetyl-β-D-glucosaminidase, α1-microglobulin, and neutrophil gelatinase-associated lipocalin	[38] 17 September 2021
HC (*n* = 185) HC vaccinated (*n* = 19) long-COVID-19 (*n* = 12) non-COVID-19 * (*n* = 37) COVID-19 (*n* = 46)	Metabolomics (Raman spectroscopy)	Identify changes in systemic inflammatory, immunologic, and metabolic reactions to infection, but not biomarkers. While this technique does not provide specific biomarker data, it identifies chemical families, which can guide the search for compounds using other methods	[39] 21 December 2021
HC (*n* = 102) COVID-19 (*n* = 248) - asymptomatic (*n* = 60) - moderate (*n* = 161) - critical (*n* = 27)	Metabolomics (UPLC-Q-TOF/MS)	**Diagnosis (COVID-19 vs. HC)** ↑: 2-phenylacetamide ↓: oxoglutaric acid and indoxyl **Diagnosis (Asymptomatic vs. HC)** ↑: dihydro-5-pentyl-2(3H)-furanone ↓: hypoxanthine and uric acid	[40] 22 December 2021
HC (*n* = 16) non-COVID-19 * (*n* = 3) COVID-19 (*n* = 56) - moderate (*n* = 26) - critical (*n* = 30) * Proximal tubule dysfunction (PTD)	Metabolomics (LC–MS/MS)	**Diagnosis (COVID-19 vs. HC and non-COVID-19)** ↑: kynurenine/tryptophan ratio and aminoaciduria: phenylalanine, leucine, proline, and tryptophan (and their related metabolites: kynurenine, 3-hydroxykynurenine, and 3-hydroxyanthranilate) **Severity (Critical vs. Moderate)** ↑: kynurenine/tryptophan ratio, kynurenine, and 3-hydroxykynurenine **Prognosis (Non-survivor vs. Survivors)** ↑: kynurenine	[41] 17 February 2022
HC (*n* = 16) COVID-19 (*n* = 16) - moderate (*n* = 16)	Lipidomics (LC-MS)	**Diagnosis (COVID-19 vs. HC)** ↑: Docosahexaenoic acid, arachidonic acid, and metabolites of major proinflammatory lipid mediators (PGE_2_, TXA_2_, and PGF_2α_)	[42] 11 May 2022
HC (*n* = 18) COVID-19 (*n* = 35)	Metabolomics (^1^H NMR)	**Diagnosis (COVID-19 vs. HC)** ↑: ketone bodies (acetoacetate, 2-hydroxybutyrate, and acetone) TMAO, glucose, formic acid, phenylalanine, and tyrosine ↓: citric acid, creatine/creatinine ratio, glycine, and hippuric acid	[43] 29 July 2022
HC (*n* = 36) COVID-19 (*n* = 104) COVID-19 * (*n* = 43) * AKI	Clinical analysis and Metabolomics (HPLC-MS)	**Diagnosis (COVID-19 vs. HC)** ↑ urinalysis: ACE2, TNF-RI, TNF-RII, and NGAL ↑ metabolomics: aminoaciduria: lysine, threonine, leucine, isoleucine, phenylalanine, and tryptophan (and their metabolites kynurenine and quinolinate). ↓metabolomics: glycine **Prognosis (COVID-19 with AKI vs. COVID-19)** ↑ urinalysis: ACE2 and TNF-RI	[44] 30 July 2022
HC (*n* = 95) COVID-19 (*n* = 91) - mild (*n* = 25) - moderate (*n* = 45) - critical (*n* = 41)	Lipidomics (LC-MS/MS)	**Diagnosis (COVID-19 vs. HC)** ↑: phosphatidylserine, lysophosphatidylserine, phosphatidylethanolamine, and lysophosphatidylethanolamine ↓:C18:0, C18:1, C22:0, and C24:0 ceramides, sphingosine, dihydrosphingosine, phosphatidylcholine, lysophosphatidylcholine, lysophosphatidic acid, and phosphatidylglycerol **Severity (Critical vs. Moderate vs. Mild)** ↑: sphingomyelin, ceramides, sphingosine, dihydrosphingosine, dihydrosphingosine l-phosphate, phosphatidylcholine, lysophosphatidic acid, phosphatidylserine, lysophosphatidylserine, phosphatidylethanolamine, lysophosphatidylethanolamine, phosphatidylglycerol, lysophosphatidylglycerol, phosphatidylinositol, and lysophosphatidylinositol	[45] 3 August 2022
HC (*n* = 104) COVID-19 (*n* = 24)	Metabolomics (IA-MS/MS)	**Diagnosis (COVID-19 vs. HC)** ↑: acetylcarnitine and C5 acylcarnitines ↓: Glycine, cysteine, valine, phenylalanine, tryptophan, C6 acylcarnitines, C8 acylcarnitines, and C16 acylcarnitines	[46] 25 August 2022
non-COVID-19 * (*n* = 17) COVID-19 * (*n* = 42) * ICU patients with acute respiratory distress syndrome (ARDS)	Metabolomics and proteomics (UPLC-MS/MS)	**Diagnosis (COVID-19 vs. non-COVID-19)** ↑ met: cyclo(pro-sulfo-tyr), 3-carboxy-4-methyl-5-propyl-2, tiglyl carnitine (C5), and furanpropanoate, uracil ↑ prot: PGLYRP1, GP6, and ARTN ↓met: 7-methylurate ↓prot: CCL23 and IGFBP-1 **Prognosis (Non-survivor vs. Survivors)** ↑ prot: CXCL16, IGFBP-2, CCL15, and CXCL9	[47] 29 September 2022
HC (*n* = 23) COVID-19 * (*n* = 60) - moderate (*n* = 26) - critical (*n* = 34) Repeated at days: 1–2, 3–4, 5–8, and weekly thereafter	Clinical analysis	**Severity (Critical vs. Moderate)** ↑: Cysteinyl leukotrienes (not at day 1–2) **Prognosis (30-Day Mortality Predicted at Admission)** ↑: Cysteinyl leukotrienes (not at day 1–2)	[48] 17 October 2022
HC (*n* = 50) COVID-19 (*n* = 126)	Metabolomics (GC-QTOF-MS)	**Diagnosis (COVID-19 vs. HC)** ↑: benzoic acid, hippuric acid, d-glucuronic acid, xylulose, myo-inositol, d-xylitol, galacturonic acid, uric acid, saccharic acid, and several amino acids: valine, leucine, isoleucine, glycine, serine, and threonine ↓: 3,4-dihydroxyphenylacetic acid, 3-hydroxypropanoic acid, and phenylalanine	[49] 24 November 2022
HC (*n* = 24) COVID-19 (*n* = 24) Repeated at days: 1, 5–8, and 29–54	Metabolomics (^1^H NMR)	**Diagnosis (COVID-19 vs. HC)** ↑: carnitine ↓: citrate, hippurate, pyruvate, and alanine **Prognosis (Day 1 vs. Day 5–8)** ↑: carnitine, hypoxanthine, acetone, and acetate **Prognosis (Day 29–54 vs. Day 5–8)** ↓: carnitine, hypoxanthine, acetone, and acetate	[50] 7 February 2023
HC (*n* = 12) ex-COVID-19 (*n* = 8)	Metabolomics (UHPLC-HRMS)	**Prognosis (ex-COVID-19 vs. HC)** ↑: L-threonine, 5-hydroxylysine, acetoacetate, 2-phenylglycine, 11*β*−13,14-dihydro-15-keto prostaglandin f2*α*, and ethyl 3-indoleacetate ↓: *N*-acetyl-L-tryptophan	[51] 22 April 2023
long-COVID-19 (*n* = 124) ex-COVID-19 (*n* = 24)	Metabolomics (GC-MS)	**Prognosis (long-COVID-19 vs. ex-COVID-19)** ↑: Guanidino acetate ↓: Advanced glycation endproduct (AGE) and *N*^G^-carboxyethylarginine (CEA)	[52] 21 June 2023
HC (*n* = 8) non-COVID-19 * (*n* = 8) long-COVID-19 (*n* = 25) * Myalgic encephalomyelitis or chronic fatigue syndrome	Metabolomics (LC-MS/MS)	**Prognosis (long-COVID-19 vs. HC)** ↓: phenylalanine and methylmalonic acid **Prognosis (long-COVID-19 vs. non-COVID-19)** ↑: quinolinic acid, kynurenine/tryptophan ratio, neopterin, and nicotinic acid	[53] 24 July 2023
HC dataset (*n* = 309) COVID-19 (*n* = 243) - mild (*n* = 186) - moderate (*n* = 57)	Metabolomics (^1^H NMR and LC-MS)	**Diagnosis (COVID-19 vs. HC)** ↑: glucose, acetone, taurine, lactic acid, acetoacetic acid, succinic acid, fumaric acid, orotic acid, caffeine, theobromine, and pantothenic acid ↓: tartaric acid, creatine, hippuric acid, allantoin, oxypurinol, trigonelline, citric acid, dimethylamine, glycine, *N*,*N*-dimethyglycine, and glycolic acid **Severity (Moderate vs. Mild)** ↑: antiviral nucleosides (ddhC, ddhC-5′Hcy, ddhC-5′CA, ddhU), phenylalanine, taurine, neopterin, glucose, 3-hydroxykynurenine, kynurenine, 3-hydroxyanthranilic acid, and quinolinic acid ↓: citric acid, trigonelline, allantoin, and glycine	[54] 12 September 2023
COVID-19 (*n* = 35) COVID-19 * (*n* = 35) * PTD	Clinical analysis	**Prognosis (COVID-19 with PTD vs. COVID-19)** ↑: AKI development	[55] 13 September 2023
HC (*n* = 57) COVID-19 (*n* = 216) - mild (*n* = 106) - moderate (*n* = 71) - critical (*n* = 39)	Transcriptomics (RT-PCR)	**Diagnosis (COVID-19 vs. HC)** ↑: miR-155, miR-let-7, and miR-223 **Severity (Mild vs. HC)** ↑: miR-21, miR-146a, and miR-155 **Severity (Moderate vs. Mild)** ↑: miR-let-7 ↓: miR-146b **Severity (Critical vs. Moderate)** ↑: miR-223	[56] 13 October 2023
COVID-19 * (*n* = 108) long-COVID-19 (*n* = 14) ex-COVID-19 (*n* = 8) Repeated at days: 1–4 and 4–7	Metabolomics (HPLC-FLD/PDA and UHPLC-MS/MS)	**Prognosis (long-COVID-19 vs. ex-COVID-19)** ↑: neopterin, kynurenine, and kynurenine/tryptophan ratio **Prognosis (Non-survivor vs. Survivors)** ↑: kynurenine/tryptophan ratio ↓: tryptophan/creatinine ratio	[57] 15 November 2023
HC (*n* = 45) COVID-19 (*n* = 119) - mild (*n* = 67) - moderate (*n* = 42) - critical (*n* = 10)	Clinical analysis	**Diagnosis (COVID-19 vs. HC)** ↑: occult blood, proteinuria, and pH ↓: specific gravity **Severity (Moderate vs. Mild)** ↑: glucose and proteinuria **Severity (Critical vs. Moderate)** ↑: proteinuria ↓: glucose	[58] 16 January 2024
HC (*n* = 16) ex-COVID-19 (*n* = 16)	Metabolomics (UPLC-MS/MS)	**Prognosis (ex-COVID-19 vs. HC)** ↑: pseudoephedrine and resveratrol sulfate ↓: cis-urocanate	[59] 5 March 2024
HC (*n* = 32) COVID-19 (*n* = 80) - mild (*n* = 46) - critical (*n* = 34)	Metabolomics (^1^H NMR)	**Diagnosis (COVID-19 vs. HC)** ↑: methionine, fucose, lysine, hippurate, 2-phenylpropionate, glutamine, 3-indoxylsulphate, and pseudouridine ↓: formate, 3-methylhistidine, trigonelline, and creatinine **Severity (Critical vs. Mild)** ↑: TMAO, hippurate, urea, dimethylamine, 3-hydroxybutyrate, fucose, 4-hydroxyphenyl acetate, 3-methyl-2-oxovalerate, 3-indoxylsulphate, tryptophan, and formate **Prognosis (Dead vs. Survivors)** ↑: creatine and hippurate ↓: cis-aconitate, urea, formate, creatinine, methanol, dimethylamine, and TMAO	[60] 30 May 2024
HC (*n* = 6) non-COVID-19 * (*n* = 12) COVID-19 (*n* = 18) * Kidney transplant recipients with albuminuria	Clinical analysis	**Diagnosis (COVID-19 vs. HC)** ↑: ACE2, albumin/creatinine ratio **Diagnosis (COVID-19 vs. non-COVID-19)** ↑: ACE2 compared ↓: albumin/creatinine ratio (12 times lower) **Diagnosis (non-COVID-19 vs. HC)** ↑: albumin/creatinine ratio (100 times higher) ↓: ACE2	[61] 10 June 2024
non-COVID-19 * (*n* = 64) COVID-19 * (*n* = 117) * ICU	Clinical analysis	**Prognosis (COVID-19 vs. non-COVID-19)** ↓: EGF (increased 7%/day in the non-COVID-19 group but only 0.5%/day in COVID-19 group)	[62] 26 June 2024

**Table 2:** Selected potential biomarkers for diagnosis.

Biomarker	Effect	Relevance
Citric acid	Reduced [29,32,43,50,54]	A marker of mitochondrial dysfunction and metabolic stress, useful for identifying early states of hypoxia or energy disruption
Proteins	Increased [23,29,36,58]	Proteinuria is an early indicator of renal dysfunction, showing elevated levels compared to healthy controls but reduced levels in patients with acute renal injuries [38]
Amino acids	Increased [23,41,44]	Aminoaciduria reflects more advanced tubular damage compared to proteinuria. Other studies also highlight the increased excretion of amino acids, such as leucine, isoleucine and threonine [41,44,49], lysine [44,60], and tyrosine [34,43]
ACE2	Increased [37,44,61]	A direct marker of viral impact on target organs, potentially correlating with viral load and initial disease severity. Elevated levels also have been observed in patients with pneumonia [37] and those with renal impairment [61]
Ketone bodies	Increased [22,43,54]	An early marker of metabolic stress, easily detectable but with limited specificity, as levels can be influenced by other conditions, such as diabetes. It is also statistically significant in pregnant individuals [22]
Kynurenine	Increased [29,41,44]	A marker of immune activation and systemic inflammation, potentially associated with progressive immunosuppression. Levels are also elevated in COVID-19 patients compared to those with proximal tubule dysfunction [41]

**Table 3:** Selected potential biomarkers for severity.

Biomarker	Effect	Relevance
Glucose	Increased [20,21,58]	Glucosuria is an indicator of severe metabolic stress. However, it is important to note that it is commonly observed in patients with preexisting diabetes, a group that is more susceptible to developing severe forms of COVID-19
Proteins	Increased [20,36,58]	Proteinuria is also used as a diagnostic biomarker, but its levels increase progressively with the severity of COVID-19, making it a valuable tool for monitoring disease progression
Red blood cells	Increased [20,21]	Hematuria reflects severe structural renal damage and is associated with the progression toward serious complications
Ketone bodies	Increased [21,60]	It serves as an indicator of metabolic stress and reflects the body’s adaptation to hypoxia and glucose depletion

**Table 4:** Selected potential biomarkers for prognosis.

Biomarker	Effect	Relevance
Kynurenine and KTR	Increased [34,41,53,57]	A key indicator of persistent inflammation and oxidative stress, associated with the development of AKI [34], mortality [41,57], and the onset of long COVID [53,57]
Neopterin	Increased [53,57]	An indicator of persistent inflammation and immune activation, with elevated levels linked to an increased likelihood of mortality [57] and the development of long COVID-19 [53,57] following the acute phase of the disease
Hypoxanthine	Decreased [34,50]	Patients with a worse prognosis exhibit low levels during the initial weeks [34,50], along with a higher likelihood of developing AKI [34]. Subsequently, the relationship reverses, with those having a worse prognosis showing elevated levels later in the disease course [50]
Ketone bodies	Increased [50,51]	Patients with a worse prognosis exhibit elevated levels during the initial weeks [50]. Later, the relationship reverses, with those having a worse prognosis showing lower levels [50]. Levels remain elevated in recovered patients [51]

## Data Availability

Not applicable.

## Appendix A

The following abbreviations are used in this manuscript:

### Appendix A.1. Population Nomenclature

HC: individuals without COVID-19 or any other pathology; non-COVID-19: patients without COVID-19 but with another pathology; ex-COVID-19: recovered COVID-19 patients; long COVID-19: patients with persistent COVID-19 symptoms weeks or months after the initial infection; COVID-19 critical: WHO stages 6–8 [10]; COVID-19 moderate: WHO stages 4–5 [10]; COVID-19 mild: WHO stages 1–3 [10]; and COVID-19 asymptomatic: COVID-19 patients without any symptoms.

### Appendix A.2. Population Abbreviations

AKI: acute kidney injury; ARDS: acute respiratory distress syndrome; ICU: intensive care unit; PTD: proximal tubular dysfunction; and WHO: World Health Organization.

### Appendix A.3. Approach and Analytical Platform Abbreviations

^1^H NMR: Proton Nuclear Magnetic Resonance; CE-MS: capillary electrophoresis-mass spectrometry; ddPCR: digital droplet polymerase chain reaction; DSI-MS: desorption electrospray ionization mass spectrometry; GC-MS: gas chromatography-mass spectrometry; GC-QTOF-MS: gas chromatography-quadrupole time-of-flight mass spectrometry; HPLC MS/MS: high-performance liquid chromatography-tandem mass spectrometry; HPLC-FLD/PDA: high-performance liquid chromatography-fluorescence/photodiode array detection; HPLC-MS: high-performance liquid chromatography-mass spectrometry; IA-MS/MS: immunoassay-mass spectrometry; LC-MS/MS: liquid chromatography-tandem mass spectrometry; UHPLC-HRMS: ultra high-performance liquid chromatography-high resolution mass spectrometry; UPLC-MS/MS: ultra performance liquid chromatography-tandem mass spectrometry; UPLC-Q-TOF/MS: ultra performance liquid chromatography-quadrupole time-of-flight mass spectrometry.

### Appendix A.4. Results and Potential Biomarkers Abbreviations

ACE2: angiotensin-converting enzyme 2; AGE: advanced glycation endproduct; ALDOB: aldolase B; APOA1, APOA5: apolipoprotein A1, A5; APP: amyloid beta precursor protein; ARTN: artemin; C3, C4B: complement component 3, 4B; CAIX: Carbonic Anhydrase IX; CCL14, CCL15, CCL23: C-C motif chemokine ligand 14, 15, 23; CD9, CD14, CD99: cluster of differentiation 9, 14, 99; CEA: NG-Carboxyethylarginine; CLEC3B: C-type lectin domain family 3 member B; COPB1: coatomer protein complex subunit beta 1; CPM: carboxypeptidase M; CTNNB1: catenin beta 1; CUBN: cubilin; CXCL9, CXCL14, CXCL16: C-X-C motif chemokine ligands 9, 14, 16; cyclic AMP: cyclic adenosine monophosphate; ddhC: dideoxycytidine; ddhC-5′CA: ddhC-5′-cysteinylalanine; ddhC-5′Hcy: ddhC-5′-homocysteine; ddhU: Dideoxyuridine; DDOST: dolichyl-diphosphooligosaccharide–protein glycosyltransferase subunit; DDX58: DEAD-box helicase 58; EGF: epidermal growth factor; EHHADH: enoyl-CoA hydratase and 3-hydroxyacyl CoA dehydrogenase; EPHB2, EPHB3, EPHB4, EPHB6: ephrin type-B receptors 2, 3, 4, 6; ESCRT: endosomal sorting complex required for transport; FABP1: fatty acid binding protein 1; FGA: fibrinogen alpha chain; FKBP1A: FK506 binding protein 1A; Gal-9: galectin-9; GLB1: galactosidase beta 1; GMFG: glia maturation factor gamma; GNS: glucosamine (N-acetyl)-6-sulfatase; GP6: glycoprotein VI; GPX4: glutathione peroxidase 4; HGF: hepatocyte growth factor; HO-1: heme oxygenase 1; HSPG2: heparan sulfate proteoglycan 2; HYOU1: hypoxia up-regulated 1; IDO1: indoleamine 2,3-dioxygenase 1; IFN-γ: interferon-gamma; IGFBP-1, IGFBP-2: insulin-like growth factor binding protein 1, and 2; IGLV1–40, IGLV1–44: Immunoglobulin Lambda Variable 1–40, and 1–44; IL-2, IL-6, IL-8, IL-12, IL-15, IL-18, IL-34: interleukins 2, 6, 8, 12, 15, 18, and 34; INR: international normalized ratio; KIM-1: kidney injury molecule-1; KTR: kynurenine/tryptophan ratio; LAG-3: lymphocyte activation gene 3; LGALS3BP: galectin 3 binding protein; LIPG: lipase G, endothelial type; LMNA: Lamin A/C; LRP2: low-density lipoprotein receptor-related protein 2; MCP-1, MCP-3: monocyte chemoattractant protein 1, and 3; miR-21, miR-146a, miR-155, miR-223, miR-2392, miR-let-7: MicroRNA 21, 146a, 155, 223, 2392, and let-7 family; MME: membrane metallo-endopeptidase; MUC-16: Mucin 16; MYD88: myeloid differentiation primary response 88; NAD+: nicotinamide adenine dinucleotide (oxidized form); NAGLU: alpha-N-acetylglucosaminidase; NAPSA: napsin A aspartic peptidase; NCK1: NCK adaptor protein 1; NGAL: neutrophil gelatinase-associated lipocalin; NPC2: Niemann–Pick disease, type C2; PARK7: Parkinsonism associated deglycase; PD-L1: programmed death-ligand 1; PGE2, PGF2α: prostaglandin E2, and F2-alpha; PGK1: phosphoglycerate kinase 1; PGLYRP1: peptidoglycan recognition protein 1; PIGR: polymeric immunoglobulin receptor; PSAP: prosaposin; PSMD12: proteasome 26S subunit, non-ATPase 12; RAC1: Ras-related C3 botulinum toxin substrate 1; RAMP3: receptor activity-modifying protein 3; RBP4: retinol-binding protein 4; RHOA: Ras homolog family member A; RIG-I: retinoic acid-inducible gene I; ROS: reactive oxygen species; RT-PCR: Reverse Transcription Polymerase Chain Reaction; S100-A9: calprotectin; SAA2: serum amyloid A2; SERPINA1: serpin family A member 1; SERPINB6: serpin family B member 6; SERPINC1: serpin family C member 1; SIPR3: sphingosine-1-phosphate receptor 3; SOD1, SOD3: superoxide dismutase 1, and 3; SPON2: spondin 2; STAT1, STAT3: signal transducer and activator of transcription 1, and 3; TCA cycle: Tricarboxylic Acid Cycle; TGFB1: transforming growth factor beta 1; TMAO: trimethylamine N-oxide; TNF-RI, TNF-RII: Tumor Necrosis Factor Receptor I, and II; TNFRS12A, TNFRS21: Tumor Necrosis Factor Receptor Superfamily Member 12A, and 21; TPI1: triosephosphate isomerase 1; TTR: transthyretin; TXA2: thromboxane A2; TXNDC17: thioredoxin domain containing 17; VEGFA: vascular endothelial growth factor A; XRCC5: X-ray repair cross-complementing protein 5; YWHAZ: tyrosine 3-monooxygenase/tryptophan 5-monooxygenase activation protein zeta.
